# ENO1 and Cancer

**DOI:** 10.1016/j.omto.2021.12.026

**Published:** 2022-01-03

**Authors:** Chen Kai Huang, Ying Sun, Lei Lv, Yong Ping

**Affiliations:** 1 Department of Molecular and Cellular Biology, University of California, Berkeley, 110 Sproul Hall, Berkeley, CA 94720, USA; 2 Department of Immunology and Microbiology, Shanghai Jiao Tong University School of Medicine, South Chongqing Road 225, Shanghai, China; 3 MOE Key Laboratory of Metabolism and Molecular Medicine, Department of Biochemistry and Molecular Biology, School of Basic Medical Sciences, Fudan University, Shanghai, 200032, China; 4 Bio-X Institutes, Key Laboratory for the Genetics of Developmental and Neuropsychiatric Disorders (Ministry of Education), Shanghai Jiao Tong University, Shanghai 200240, China

**Keywords:** ENO1, hallmarks of cancer, glycolysis, cancer biomarker, tumorigenesis, oncotherapy

## Abstract

α-Enolase (ENO1), also known as 2-phospho-D-glycerate hydrolase, is a glycolytic enzyme that catalyzes the conversion of 2-phosphoglyceric acid to phosphoenolpyruvic acid during glycolysis. It is a multifunctional oncoprotein that is present both in cell surface and cytoplasm, contributing to hit seven out of ten “hallmarks of cancer.” ENO1's glycolytic function deregulates cellular energetic, sustains tumor proliferation, and inhibits cancer cell apoptosis. Moreover, ENO1 evades growth suppressors and helps tumors to avoid immune destruction. Besides, ENO1 “moonlights” on the cell surface and acts as a plasminogen receptor, promoting cancer invasion and metastasis by inducing angiogenesis. Overexpression of ENO1 on a myriad of cancer types together with its localization on the tumor surface makes it a great prognostic and diagnostic cancer biomarker as well as an accessible oncotherapeutic target. This review summarizes the up-to-date knowledge about the relationship between ENO1 and cancer, examines ENO1's potential as a cancer biomarker, and discusses ENO1's role in novel onco-immunotherapeutic strategies.

## Graphical abstract

**Figure:**
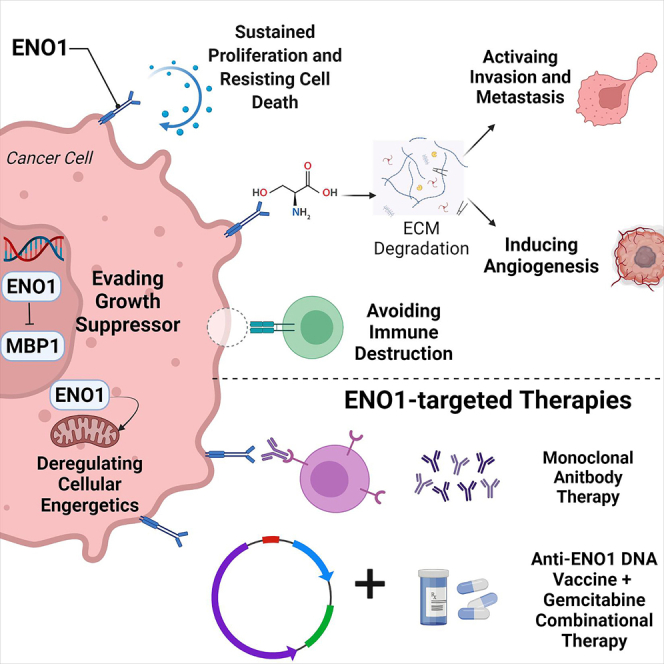

## Teaser

Ping and colleagues review the relationship between ENO1 and multiple cancer types, evaluate ENO1's potential role in tumor development, and update recent advancements in ENO1-targeted onco-therapies.

## Introduction

Alteration in glucose metabolism is the best-known example of metabolic reprogramming in cancer cells. Metabolic reprogramming is one of the hallmarks of cancer cells. The peculiar tendency of cancer cells to choose the relatively energy-deficient aerobic glycolytic pathway (Warburg effect) over the energy-efficient oxidative phosphorylation pathway attracts much attention in oncology. Cancer cells take advantage of this metabolic reprogramming to drive tumor development in three stages. First, an elevated level of glucose transportation via increased expression of glucose transporters on the cell membrane such as Glut1/Glut3 enhances the glucose consumption in cancer cells. Then, metabolic intermediates produced by glucose metabolism promote the biosynthesis of nucleotides, amino acids, and triglycerides, which are essential for cell proliferation. In this process, many key metabolic enzymes become uncontrolled. For example, hexokinase, fructose 1,6-diphosphate phosphofructokinase, kinase muscle isomer 2, and pyruvate α-enolase (ENO1) have all been found to be overexpressed or overactivated in cancer cells. In the third stage, the overproduced lactate helps to create a more acidic microenvironment, which is unfavorable for the surrounding immune cells. In this scenario, enzymes that are involved in tumor glycolysis are promising targets and might open brand-new therapeutic opportunities for cancer treatment. ENO1, one of the critical enzymes in the glycolytic pathway, is a multifunctional protein with oncogenic properties: it promotes tumor cell proliferation, migration, and invasion, which result in the accelerated progression of various tumors. Among the four subtypes of enolase, ENO1 is ubiquitously expressed in most human tissues and overexpressed in a myriad of cancer types.^1^ ENO2 (β-enolase), also known as neuron-specific enolase, is predominantly expressed in neurons and neuroendocrine-related tumors.^2^^,^^3^ ENO3 (**γ**-enolase) exists primarily in muscle tissues.^4^ ENO4 (64306537H0Rik) contributes to the normal assembly of the fibrous sheath in the principal piece of the sperm flagellum.^5^ All these isoforms are highly conserved in humans, implicating their significance in cells. They play salient roles in catalyzing the penultimate step of glycolysis, which dehydrates 2-phosphoglycerate to phosphoenolpyruvate.^6^ In particular, ENO1 accounts for 90% of total cellular enolase activity in the glycolytic pathway.^7^ Moreover, ENO1 has certain tumor gene characteristics and is abnormally expressed in many human tumors, which is regulated by multiple mechanisms. Therefore, this review mainly focuses on ENO1. Taken together, ENO1 is a crucial molecular target for tumor therapy, which has emerged to be a research hotspot in recent decades. However, up to now, no review has yet systematically summarized the important functions and mechanisms of ENO1 in tumorigenesis and tumor immunology. This review summarizes the relationship between ENO1 and hallmarks of cancer, elucidates the regulators and post-translational modifications of ENO1, as well as explaining the therapeutic applications of ENO1. Special attention is focused on the role of ENO1 in tumor progression, the prospects of ENO1 as a cancer biomarker, and novel ideas in cancer treatments.

## Overview of ENO1 and its role in cancer

**Figure 1:**
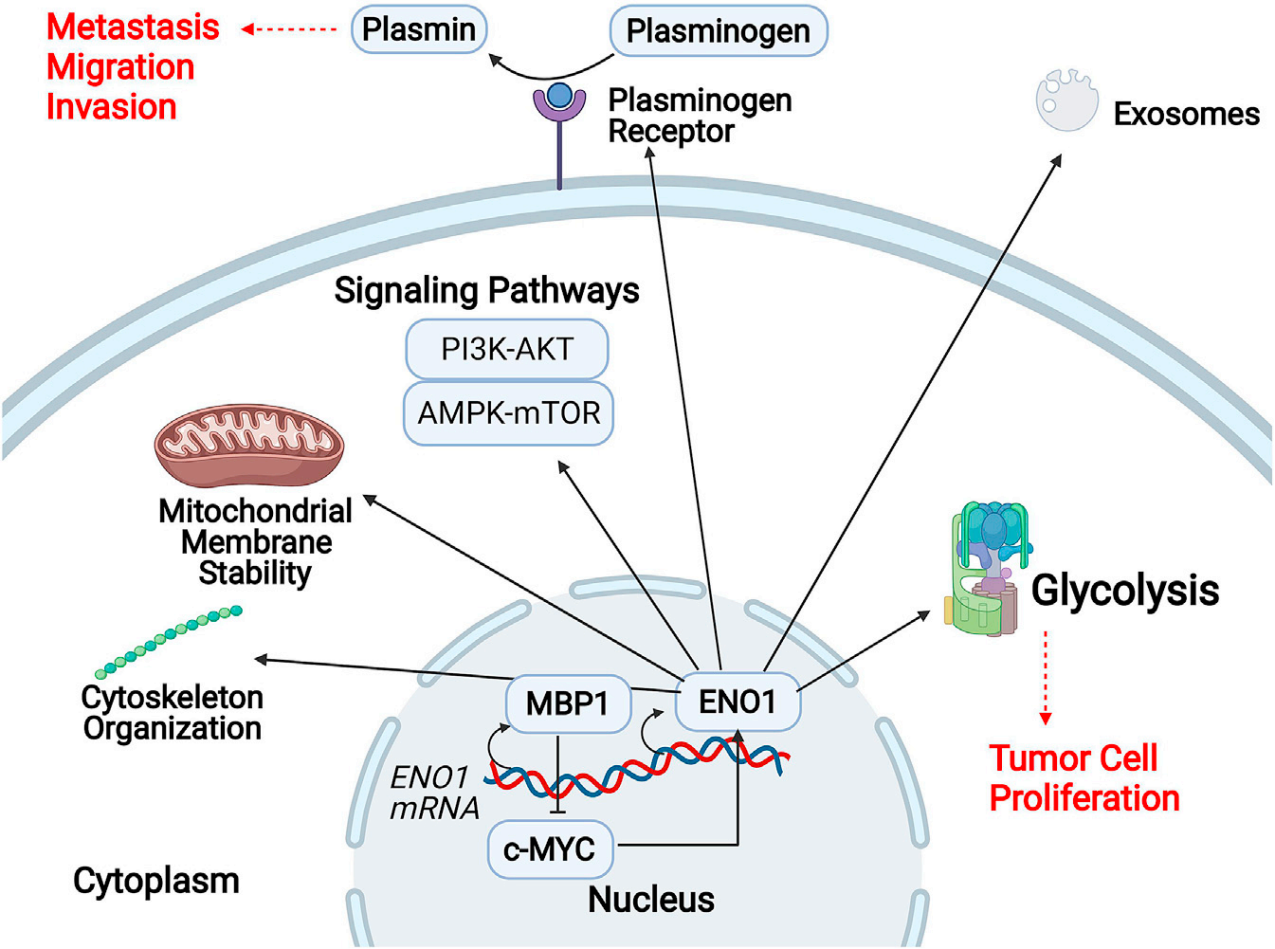
Schematic representation of ENO1's multiple cellular functions, varied by its localization In the nucleus, MBP1 (c-MYC promoter binding protein), the alternative translation variant of ENO1, suppresses c-MYC and promotes the transcriptional level of ENO1 mRNA. The cytoplasm is the major expression site of ENO1, where ENO1 catalyzes glycolysis (exploited by cancer to promote tumor cell proliferation), maintains mitochondrial membrane stability, regulates signaling pathways, and reorganizes the cytoskeleton. When localized on the cell surface, ENO1 serves as a plasminogen receptor, which converts plasminogen into plasmin (exploited by cancer to promote metastasis, migration, and invasion). In addition, ENO1 can also partake components of exosomes.

ENO1 is a multifunctional protein that partakes in various key intracellular and extracellular activities, depending on its localization (Figure 1). The primary function of ENO1 is to catalyze glycolysis; in particular, K228, K335, and K343 are the key residues that mediate ENO1 catalytic activity.^8^ When localized on the cell surface, ENO1 acts as a plasminogen receptor and promotes extracellular matrix (ECM) degradation. At the centrosome, ENO1 helps organize microtubules and the interphase cytoskeleton. In the extracellular space, ENO1 can either associate with exosomes or be secreted as a soluble protein.^9^ In the cytoplasm, ENO1 maintains mitochondrial membrane stability.^10^ Moreover, it can also regulate multiple intracellular signaling pathways.^11^

While these functions are critical to the survival of a healthy cell, genes of the glycolysis pathway, especially ENO1, have been found to overexpress in more than 70% of total human cancer cases worldwide.^12^, ^13^, ^14^, ^15^, ^16^ For example, in lung cancer, protein ENO1 overexpression is associated with worse clinical outcomes. In head and neck cancer, mRNA expression of ENO1 was increased in 68% of tumor specimens when compared with their normal counterpart.^17^ In pancreatic cancer, the expression level of ENO1 positively correlates to clinical stage, lymph node metastasis, and poor prognosis.^18^^,^^19^ In colorectal cancer (CRC), overexpression of ENO1 promotes tumorigenesis and metastasis by regulating the AMPK/mTOR pathway.^20^ Conversely, tumor activities can be constrained by ENO1 knockdown,^15^^,^^21^, ^22^, ^23^, ^24^, ^25^ silencing ENO1,^26^, ^27^, ^28^ modifying microRNA,^29^ targeting long non-coding RNA,^30^ or treatment with anti-ENO1 autoantibodies (Abs).^31^^,^^32^

There are three fundamental qualities of ENO1 that make it an excellent cancer biomarker: firstly, cell surface localization makes it accessible for imaging and treatment; secondly, overexpression in cancer cells versus lower expression in normal cells; thirdly, overexpression positively correlates with worse prognosis and clinical outcomes. Across a broad spectrum of tumors, both the diagnostic and prognostic value of ENO1 overexpression has been proven. In lung cancer, patients with overexpressed ENO1 tend to show poor clinical results, with shortened overall and progression-free survival.^13^^,^^31^ In breast cancer, ENO1 overexpression correlates with larger tumor size and poor nodal status.^33^ In hepatocellular carcinoma, enhanced ENO1 expression increased with tumor metastasis and correlated positively with venous invasion.^34^^,^^35^ Taking into consideration the correlation between ENO1 overexpression and worse cancer prognosis, ENO1 stands out as a discriminatory cancer biomarker (Table 1).

**Table 1:** Overexpression of ENO1 in cancers and its clinical correlations

Cancer type	ENO1 overexpression	Clinical correlation	References
Breast	mRNA & protein	disease progression, poor distant metastasis-free survival, overall survival, chemoresistance, radiation resistance	Tsu et al.,^33^ Zhang et al.,^45^ Gao et al.,^58^ Cancemi et al.,^63^
Colorectal	mRNA & protein	disease progression, overall survival	Zhan et al.,^20^ Liu et al.,^77^
Eye	mRNA & protein	disease progression, overall survival	Liu et al.,^29^
Gastric	mRNA & protein	disease progression, overall survival, chemoresistance	Sun et al.,^15^, ^21^, ^22^, ^23^, ^24^, ^25^ Qiao et al.,^46^
Glioma	mRNA & protein	disease progression	Song et al.,^23^
Head and neck	mRNA & protein	overall survival, poor disease-free survival	Tsai et al.,^17^
Leukemia	protein	disease progression	Griggio et al.,^40^
Liver	mRNA & protein	disease progression	Yu et al.,^30^ Takashima et al.,^34^, ^35^ Hamaguchi et al.,^34^, ^35^
Lung	mRNA & protein	disease progression	Chang et al.,^13^ He et al.,^14^ Fu et al.,^15^ Zhang et al.,^16^ Almaguel et al.,^43^ Chen et al.,^47^
Pancreas	mRNA & protein	disease progression, overall survival, chemoresistance	Yin et al.,^19^ Principe et al.,^26^^,^^32^ Wang et al.,^27^ Tomaino et al.,^37^ Wang et al.,^84^ Peng et al.,^86^ Cappello et al.,^87^^,^^88^ Di Caro et al.,^89^
Prostrate	mRNA & protein	disease progression, chemoresistance	Satani et al.,^83^
Skin	protein	disease progression	Zhang et al.,^45^ De Rosa et al.,^73^

In addition, anti-ENO1 Ab is also a competent indicator of clinical outcomes in various types of cancer.^36^, ^37^, ^38^, ^39^, ^40^ In search of more precise detection results, a panel of four cancer biomarkers was employed, including cancer antigen 125, carcinoembryonic antigen, annexin A1 Abs, and anti-ENO1 Abs for upgraded diagnostic ability.^41^ Results revealed that the diagnostic performance was significantly enhanced through their combination of two Abs: annexin A1-Ab and ENO1-Ab. Also utilizing a panel of biomarkers for cancer immunodiagnosis, Dai et al.^42^ combined anti-ENO1 with two other tumor protein biomarkers (carcinoembryonic antigen and cytokeratin 19); consequently, the sensitivity in the diagnosis of the lung was increased to 84%. In summary, ENO1 is expected to be a useful cancer biomarker for guiding patient management and changing disease timelines. In the near future, ENO1 surface imaging will be utilized to screen for tumors that have yet to be scrutinized. These data might be used to enhance the prognosis and management of patients by tracking disease development, detecting recurrence, and assessing prognosis.^43^

## ENO1 in tumorigenesis and oncogenic mechanism

### ENO1 and hallmarks of cancer

In the effort to organize principles for rationalizing the complexities of neoplastic disease, two distinguished professors, Hanahan and Weinberg,^44^ together coined the idea of “hallmarks of cancer” based on biological features that tumors acquired during the multistep developments of human tumors. Studies have confirmed that ENO1 is overexpressed in many tumors and contributes to seven out of the ten hallmarks of cancer: (1) sustaining proliferative signaling, (2) activating invasion and metastasis, (3) inducing angiogenesis, (4) evading growth suppressors, and (5) resisting cell death. More intriguingly, ENO1 also shows new tumor propensities not summarized in Hanahan and Weinberg's 2000 publication: (6) deregulating cellular energetic and (7) avoiding immune destruction. Although ENO1 is regarded as an emerging tumor biomarker and oncotherapeutic target, the specific mechanisms of how ENO1 regulates tumor progression have not yet been comprehensively determined. Here, we review up-to-date articles and summarize the new findings that shed fresh insight on the relationship between ENO1 and tumor progression.

#### ENO1 sustains tumor proliferation and resists cancer cell death

The occurrence of tumors is related to the non-stop proliferation and abnormal apoptosis of cells. Numerous studies have found that ENO1 can promote proliferation and inhibit apoptosis in various types of malignant tumors cells. In SK-BR-3 breast cancer, silencing of ENO1 caused a significant decline in tumor proliferation and colony formation. Cells were blocked in the G2/M phase, with significantly reduced expression levels of cell-cycle-related proteins, such as DC25, CDC2, and cyclin B1. At the same time, the silencing of ENO1 substantially elevated the rate of cancer cell apoptosis by inducing the upregulation of apoptosis-related protein Bax and the downregulation of Bcl-2. In addition, the phosphorylation levels of PI3K and AKT were also significantly reduced, suggesting that ENO1 may affect tumor cell activity by mediating the PI3K/AKT pathway and the corresponding downstream signaling pathways.^45^ Another article also demonstrated that, in gastric cancer, knockdown of ENO1 led to the arrest of the cell cycle at the G1 phase and promoted the apoptosis of MKN-45 and MGC-803 cells.^46^ Furthermore, mechanistic analyses revealed that ENO1 enhanced cell proliferation by accelerating G1 progression and upregulating G1 phase cyclin-dependent kinase 6. The improvement of cell survival by ENO1 was by upregulating p38 in the MAPK cascade and increasing p-AKT in the AKT cascade, in particular in lung cancer cell lines.^47^ However, not all overexpression of ENO1 promoted tumor proliferation and inhibited apoptosis. Most tumor cells show the Warburg effect, reprogramming the metabolic processes associated with increased glycolytic enzyme levels to enhance the glycolytic flux, but some other tumor cells exist that do not possess this phenomenon. In neuroblastoma, the upregulation of ENO1 expression can inhibit cell proliferation and induce apoptosis, and the ENO1 gene has a strong dose-dependent inhibitory effect on the growth of tumor cells, indicating that the significance of ENO1 is varied in different types of tumor cells.^48^ This diversification may be tumor specific and related to the subcellular localization of ENO1.

#### ENO1 promotes invasion and metastasis of tumor cells

Multiple studies have shown that overexpression of ENO1 positively correlates to invasion, migration, and metastasis in various types of cancers.^15^^,^^20^^,^^26^^,^^45^ The intercellular, proteolytic, and invasive activities of ENO1 are largely due to its role as a plasminogen receptor, which expedites the binding of plasminogen. Plasminogen is then converted into serine protease plasmin. Under inflammatory conditions, plasmin activation induces fibrinolysis and ECM degradation, which is the major driving force of cancer cell migration and metastasis.^31^^,^^49^ Thus, the ENO1-involved cell surface plasminogen functions are often exploited by bacteria and other pathogens to invade host tissues.^50^ Upon ENO1 silencing, the migration rate is reduced in two ways. On the cell surfaces, since ENO1, along with urokinase-type plasminogen activator, integrins, and cytoskeletons, are responsible for cell adhesion, depletion of ENO1 limits the migration ability of cancer cells.^51^ In the cytoplasm, ENO1 is a key glycolytic enzyme in the production of ATP (adenosine triphosphate), thus its depletion would demobilize tumor activities. An experimental therapy employing retinoic acid, which downregulates ENO1, significantly reduced the motility rate in follicular thyroid carcinoma cells.^52^ Conversely, elevated ENO1 expression on the cell surface causes paclitaxel-treated cancer cells to be super-invasive in breast cancer cells.^53^

Furthermore, ENO1-induced epithelial-to-mesenchymal transition (EMT), whereby epithelial cells are transformed into mesenchymal cells, also activates tumor metastasis. During EMT, epithelial cells lose their polarity and cell-cell adhesions but yield the ability to migrate, proliferate, and differentiate into specific tissues and organs. These attributes of mesenchymal cells, along with their propensity to develop chemoresistance and stem cell-like qualities, make EMT a critical factor in cancer progression.^54^ Recent studies have found that ENO1 participates in the EMT-regulating process, tumor infiltration, and metastasis in malignant epithelial tissues in lung cancer, gastric cancer, and endometrial cancer.^46^^,^^47^

The loss of E-cadherin and overexpression of mesenchymal cell markers, such as N-cadherin and vimentin, are all hallmarks of EMT. In glioma cells, knockdown of ENO1 resulted in the restoration of E-cadherin expression and suppression of vimentin expression. Therefore, ENO1 suppression can inactivate EMT progression.^23^ In reverse, under a high-glucose environment, the upregulation of hyperglycemia-induced ENO1 can inhibit the expression of E-cadherin and simultaneously enhance the expression of Snail, vimentin, and N-cadherin.^55^ In endometrial cancer, ENO1 silencing mediated the inactivation of PI3K/AKT signaling and its downstream signal suppression, including glycolysis, cell-cycle progression, and EMT-associated genes, by modulating p85.^56^ Together, these data suggest that ENO1 can affect tumor progression by mediating the PI3K/AKT oncogenic pathway and EMT development.

#### ENO1 induces angiogenesis

Tumor angiogenesis is defined as the proliferation of a network of blood vessels that supplies tumors with microenvironments enriched with oxygen and nutrients. If there is no neovascularization, the tumor diameter would not exceed 2 mm; it would remain dormant or degenerate and even disappear without metastasis.^57^ The activation of the ENO1-mediated glycolytic pathway upregulates the transcription and inhibits the apoptosis of the cellular oncogene, resulting in angiogenesis and affecting the response to hypoxia in tumor cells. In a study of breast cancer, a xenograft model was established by injecting breast cancer cells with small interfering RNA (siRNA) ENO1 into nude mice; reduced angiogenesis in the tumor tissue was observed with declined tumor growth and diminished tumor volume and weight. Under hypoxic conditions, the proliferative ability significantly decreased in the cells transfected with ENO1 siRNA. The number of cells in the G1 phase increased while it was reduced in the S phase. The findings suggest that the inhibition of ENO1 expression increases tolerance to hypoxia in tumor cells, which demonstrate slower cell growth, increased apoptosis, and a smaller tumor size.^58^ Recent studies on glioma also confirmed that silenced ENO1 expression can significantly inhibit glucose intake, lactic acid production, extracellular acidification rate, and the angiogenesis ability of glioma microvascular endothelial cells. These results suggest that ENO1-mediated aerobic glycolysis can significantly promote angiogenesis of glioma.^23^ In summary, ENO1 can reduce the tolerance of tumor cells to hypoxia by mediating aerobic glycolysis, thus promoting tumor angiogenesis.

#### ENO1 evades growth suppressor

*ENO1* is a bifunctional gene that can encodes the 48-kDa multifunctional protein ENO1 and a 37-kDa DNA binding protein called c-MYC binding protein 1 (MBP1). MBP1, which is alternatively translated from the second start codon of *ENO1* transcripts, and is preferentially localized in nuclei, while *ENO1* is found in the cytoplasm and the cell surface. Despite being encoded by the same gene in vertebrates, endogenous levels of ENO1 are often higher than that of MBP1 due to the differences in translational efficiency, translational regulation, and post-translational stability.^59^

ENO1 can affect the physiological metabolism in cancer cells and is expected to be a novel pharmacological target for cancer treatment. MBP1 also exhibits a significant influence on the growth, progression, and metastasis of cancer. ENO1 and MBP1 interact with each other through the oncoprotein c-MYC, which deregulates glycolysis through the activation of several components of the glucose metabolism pathway, including the upregulation of ENO1.^60^ On the other hand, MBP1 binds to the P2 promoter of c-MYC and functions as a transcriptional repressor for c-MYC transcription under cellular stress and low glucose conditions, slowing down cell proliferation.^61^ Under hypoxia, cancer cells tend to upregulate c-MYC, which simultaneously raises the ENO1 level to promote glycolysis, and lowers the MBP1 level, to speed up cell proliferation.^62^ In breast cancer, an inverse relationship existed between MBP1 expression and ENO1 activity, where a decrease in MBP1 was associated with a poor prognosis.^63^

In the gastric cancer cell line SC-M1, the overexpression of MBP-1 suppresses cell colony formation, migration, and invasion.^64^ Therefore, MBP1 could be a potential gene therapeutic candidate against non-small cell lung cancer (NSCLC) growth.^65^ The exogenous expression of MBP1 in NSCLC cells inhibits cell proliferation and induces mitochondrial pore formation, which does not allow accumulation of cytochrome *c*. MBP1 can also induce necrosis-like cell death with more mitochondrial permeability transition in human NSCLC cells (H1299); therefore, MBP1 could be a useful therapeutic intervention tool against NSCLC tumor growth.^66^

#### ENO1 deregulates cellular energetics in tumor cells

In most tumors, the Warburg effect enhances the total glycolysis rate in both anaerobic and aerobic conditions.^67^ Other than performing oxidative phosphorylation, which is a more efficient means of generating ATP under aerobic conditions, cancer cells oddly opt for the less-efficient glycolysis pathway, even in the presence of an abundant oxygen supply. Intense tumor proliferation requires a large amount of nucleotide, lipid, and protein synthesis; thus, cancer cells reprogram metabolic pathways to capture nutrients into anabolic pathways to satisfy the need for more cellular building blocks.^68^ Thus, aerobic glycolysis confers cancer cells the advantage in competing with normal tissues for nutrients.^24^ As glycolysis lies in the heart of the Warburg effect, ENO1 plays a pivotal role in this process. In endometrial carcinoma cells, ENO1 silencing significantly reduces glycolysis and cell proliferation.^56^ The inhibition of glycolysis results in metabolic changes that increase oxidative stress-induced autophagy, fatty acid oxidation, and amino acid catabolism, which lead to less growth and senescent phenotypes of cancer cells.^24^ All in all, studies have evaluated the importance of ENO1 in sustaining the Warburg effect, hence validating its value as a therapeutic target for deterring tumor development.

The hypoxia tumor microenvironment (TME) induces the expression of the oncogene HIF-1α (hypoxia inducible factor-1α). HIF-1α is a key oncogene that regulates glucose metabolism in tumor cells and is closely related to tumor development, varying in a cell-type-specific manner. Several studies have demonstrated that the hypoxia-related HIF-1α pathway can regulate ENO1 expression, thus contributing to tumorigenesis. Liu et al.^69^ showed that ENO1 conduces thyroid carcinoma by being involved downstream of the mTOR/HIF-1α pathway and accelerating cancer progression by regulating cystatin-SN. Another study also confirmed that silencing or inhibition of ENO1 decreased pulmonary artery smooth muscle cell (PASMC) proliferation, promoted differentiation, sensitized cells to apoptosis, and restored mitochondrial respiration. Specifically, ENO1 regulated PASMC phenotype changes via the AMPK-AKT pathway.^70^ These studies provide sufficient evidence of ENO1's participation in oncogenic metabolic pathways. In the HCT116 cell line, overexpression of ENO1 promoted cell proliferation, migration, and invasion *in vitro*, as well as tumorigenesis and metastasis *in vivo*. AMPK pathway activation or mTOR pathway suppression can block these ENO1-induced alterations. These findings suggest that ENO1 is a potent promoter of CRC genesis, at least in part, through regulating the AMPK/mTOR pathway; thus, ENO1 emerges as a promising metabolic target in CRC patients.^20^

#### Avoiding ENO1-mediated immune destruction

The TME corresponds to a complex and dynamic interconnection between the ECM and malignant cells. Tumors are infiltrated with diverse adaptive and innate immune cells that can perform both pro- and anti-tumorigenic functions. As a major branch of T cells, cytotoxic CD8+ cells detect abnormal tumor antigens distributed on the cancer cell surface and target them for destruction. In the context of TME, regulatory T cells (Tregs) promote tumor development by dampening anti-tumor immune responses, whereas effector T cells promote inflammation.^71^

ENO1 plays a key role in the regulation of T cell effector functions, including T cell activation^72^ and the suppressive functions of induced Tregs.^73^ Interestingly, downregulation of ENO1 activity represses the glycolytic activity of tumor-infiltrating CD8+ lymphocytes (CD8+ TILs), leading to their functional exhaustion.^74^ This dilemma represents ENO1-targeting oncotherapies as a double-edged sword: ENO1-induced glycolysis reduction would simultaneously downregulate both host anti-tumor TILs and hostile tumor cells.

**Figure 2:**
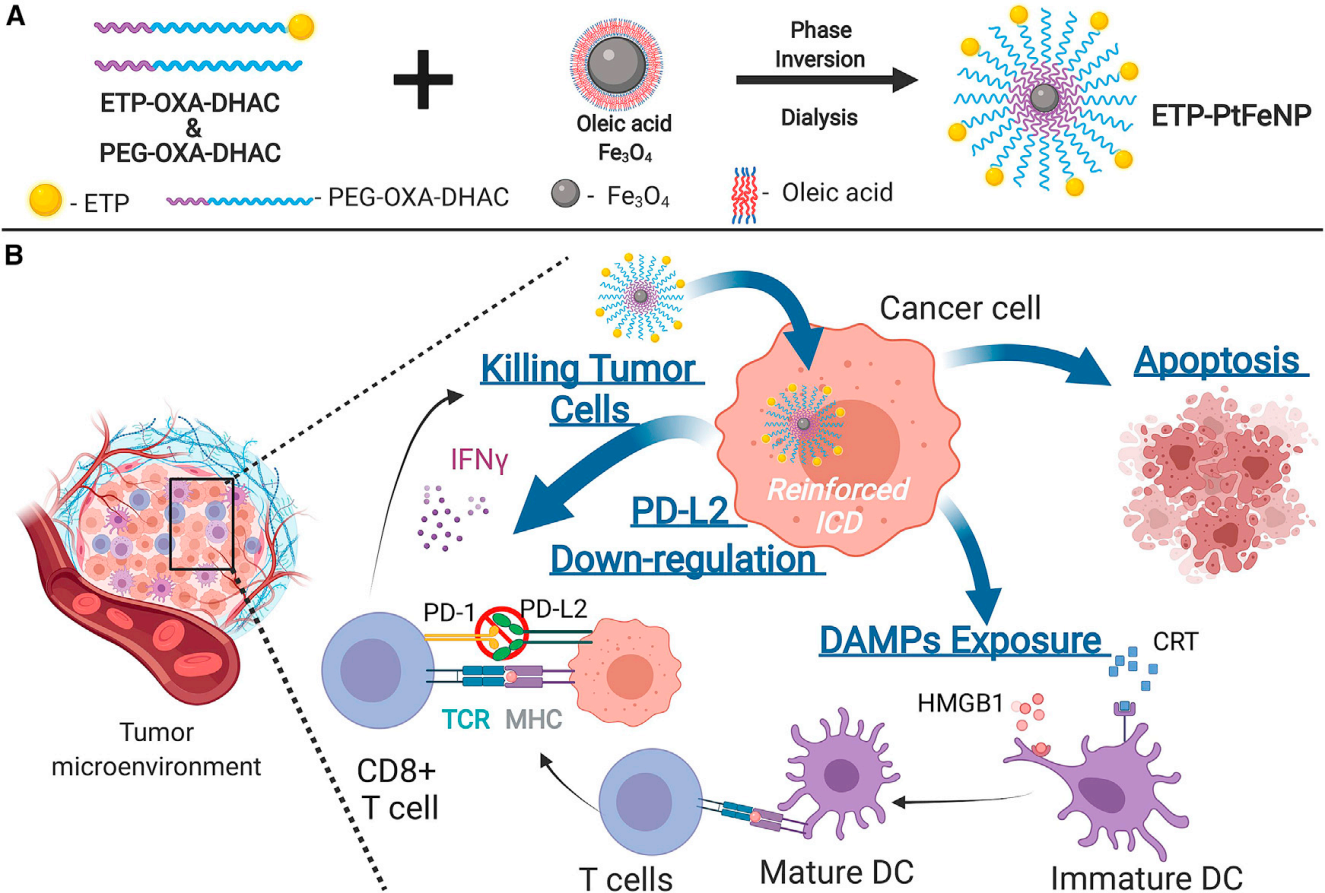
Diagram of the assembly of ETP-PtFeNP (A) ETP-OXA-DHAC (tumor-targeting polymer) and PEG-OXA-DHAC (non-targeting polymer) form the polymeric shells. The oleic acid-Fe_3_O_4_ nanoparticles were synthesized in tetrahydrofuran with modification of oleic acid. Finally, ETP-PtFeNP was obtained via the competitive binding interaction between the terminal catechol group of polymers and the carboxyl group of oleic acid using the inversion dialysis method. (B) Schematic representation of enhanced anti-tumor immune responses with ETP-PtFeNP (ENO1-targeting peptide modified Pt prodrug-loaded Fe_3_O_4_ nanoparticles) treatment. ETP-PtFeNP reinforces immunogenic cell death (ICD) in cancer cells, leading to apoptosis. ETP-PtFeNP induces damage-associated molecular patterns (DAMPs), which increases the exposure of molecules such as calreticulin (CRT) and high-mobility group B1 (HMGB1). CRT act as an engulfment signal for dendritic cells (DCs) to target cancer cells, resulting in the cross-presentation of tumor antigens and anti-tumor-specific T cell responses. HMGB1 promotes the immunogenicity of CRT by interacting with receptors on DCs. The ETP-PtFeNP formulation targets PD-L2-mediated immunosuppression on the opposite side and restores the T cell cytotoxicity. Also, ETP-PtFeNP upregulates the secretion of IFN-**γ** in tumor tissues.

Solving this predicament, Chen et al.^75^ designed a tumor-targeting core-shell magnetic nanoparticle (ETP-PtFeNP) to reinforce the immunogenic cell death induction (ICD) of loaded oxaliplatin (IV) prodrug (Figure 2). ICD's immunogenic effects are mainly regulated by the exposure of damage-associated molecular patterns, which includes surface exposure of calreticulin (CRT), ATP secretion, and high-mobility group protein B1 (HMGB1) release. In particular, CRT serves as an engulfment signal that targets apoptotic cells to DCs, leading to cross-presentation of tumor antigens and anti-tumor-specific T cell immune responses. Moreover, studies have shown that the oxaliplatin in the ETP-PtFeNP formulation could reverse PD-L2-mediated immunosuppression and reactivate T lymphocyte cytotoxicity. Within the context of TME, in addition to the unaffected properties, TILs would also increase the number of mature CD80+ CD86+ dendritic cells, cytotoxic CD8+ and helper CD4+ T cells, and effector T cells: CD4+ CD25− T cells; thus boosting the overall performance. Synergistically, ETP-PtFeNP not only induce a significant ICD but also elicit an effective anti-tumor immune response to inhibit *in vivo* tumor growth, making it a promising, multimodal agent for immunogenic chemotherapy.

Tumors thrive by avoiding immune destruction through bundling with host tissues and repressing immune cells. The lack of CD8+ and CD4+ T cells each leads to noticeable increases in tumor incidence, implicating the importance of T cells in surveillance and subsequent tumor eradication. Therefore, by sustaining T cell levels through tumor-targeted ENO1 downregulation, tumors cannot avoid being dismantled by immune responses.

### ENO1 functions regulated by non-coding RNA

In recent years, the regulation of ENO1 through non-coding RNA (ncRNA) has been attracting much attention. ncRNA is commonly employed with RNA that does not encode a protein, but this does not suggest that ncRNA does not contain information or have a function. Liu et al.^29^ performed a dual-luciferase reporter assay to confirm that miR-22-3p can directly bind to ENO1 and inhibit the proliferation of retinoblastoma cells by negatively regulating ENO1 expression. Furthermore, recent research proved that upregulation of Circ-ENO1, and its host gene ENO1, correlate to tumorigenesis in lung adenocarcinoma (LUAD) cells. Circ-ENO1 promoted glycolysis and tumor progression in LUAD through the miR-22-3p/ENO1 axis, identifying circ-ENO1 as a promising treatment target for LUAD patients.^76^ New candidates, such as circ-AMOTL1, can also promote ENO1 expression. As has been demonstrated by an *in vitro* study, ENO1 may serve as a tumor-promoting gene in oral squamous cell carcinoma through the circ-AMOTL1/miR-22-3p/miR-1294 network.^77^ Similarly, long ncRNA (lncRNA) can also directly bind to ENO1 and regulate its translation. lncRNA P5848 can upregulate both gene and protein expression levels of ENO1, promoting cancer cell growth. Conversely, siRNA-mediated knockdown of ENO1 counteracted the effects of lncRNA P5848 on cancer cell growth, cell survival, and migration. These findings provide new insights into the mechanisms underlying the lncRNA involvement in carcinogenesis and can serve as a basis for the development of novel strategies to arrest cancer progression.

### PTMs of ENO1

Post-translational modifications (PTMs) promote protein functional diversity by regulating protein activity, stability, subcellular localization, and protein-protein interactions. PTM, associated with a variety of cellular pathways and diseases, is an effective mechanism to expand gene coding and regulate cell physiological functions. Previous research has established that the PTM of ENO1 plays an essential role in basic biological processes and is closely related to tumor progression.

F box and WD40 domain protein 7 (FBXW7) is a PTM that physically binds to ENO1 and targets ENO1 for ubiquitin-mediated degradation. Functionally, FBXW7 suppresses the ENO1-induced gene expression, lactate production, cell proliferation, and migration. This suggests that ENO1 serves as a substrate of FBXW7, and its activity can be negatively regulated by FBXW7 at the post-translational level. Such a finding provides a novel molecular insight into FBXW7-directed tumor suppression through the regulation of ENO1.^78^ Similarly, Zakrzewicz et al.^79^ revealed that the methylation at arginine 50 of ENO1 (ENO1R50) assists ENO1 surface localization. More importantly, the methylated form, ENO1R50me, is found to affect cancer cell motility since the replacement of ENO1R50 by lysine diminished ENO1-triggered cell invasion. Other PTMs, such as acetylation,^80^ citrullination,^81^ and phosphorylation,^37^ also affect ENO1 immuno-activities and localization.

Considering the intriguing crosstalk between different PTMs, the combination of individual PTMs on ENO1 protein surface can create a PTM code to regulate cell behavior. This will pave a new way to design drugs for the interference of tumorigenesis and cancer progression.

## ENO1-targeting oncotherapeutic treatment

Taken together with the information mentioned above, there are three major functions of ENO1 that allow it to advance cancer progression: (1) promotion of glycolysis in cancer, (2) activation of oncogenic signaling pathways, and (3) as a driver of tumor migration, invasion, and metastasis. These attributes position ENO1 as a potent candidate to deliver targeted therapies, especially in tumors with an overexpressed level of ENO1. A myriad of studies has already provided pre-clinical data supporting the targeting basis. Therapies with ENO1 depletion effectively debilitate glycolysis, growth, proliferation, migration, metastasis, and invasion in several cancer types.^15^^,^^20^^,^^21^^,^^24^, ^25^, ^26^

Besides serving as a prognostic biomarker, anti-ENO1 Ab can also be utilized as a potent therapeutic tool to inhibit ENO1 functions. For instance, in pancreatic ductal adenocarcinoma (PDAC) patients, the circulation of anti-phosphorylated-ENO1 Ab results in longer progression-free survival and a significant reduction in the risk of death.^37^ Another study reports that, in advanced lung and breast cancer states, a distinct decrease in the anti-ENO1 Ab level is a common occurrence, implicating an improving prognosis.^36^ At first glance, these two studies seem to contradict each other; however, the truth is that the circulating anti-ENO1 Ab physically neutralizes the surface-expressed ENO1, leading to an observed reduction in the Ab level and a better prognosis. In a mouse model, anti-ENO1-specific Abs were transferred to a highly ENO1 overexpressed tumor, and the circulating anti-ENO1 Ab level was lower in the injected mice group than the control group.^39^

In addition to the antibody treatment, work has also been carried out to determine the efficacy of applying small-molecule inhibitors targeting ENO1. Jung et al.^82^ reported the first non-substrate analog of ENO1, ENOblock, which can directly bind to different isoforms of enolase and inhibits their activity. Furthermore, their analysis revealed that ENOblock can inhibit cancer cell metastasis *in vivo*. Nevertheless, the specificity of ENOblock has been challenged by Satani et al.^83^ However, the design of inhibitory therapeutic agents has never stopped. pHCT74, a novel nanoparticle-delivered peptide, targeting ENO1 in combination with doxorubicin, has been reported to display strong anti-tumor activity in pre-clinical models of prostate cancer.^84^

Chemotherapy has been a major choice for most cancer treatments. However, intrinsic or acquired drug resistance significantly reduces the clinical effectiveness of chemotherapy.^85^ Multiple studies have reported that the overexpression of ENO1 induces anti-tumor drug resistance by accelerating glycolysis,^17^^,^^27^^,^^28^ interacting with the cytoskeleton,^22^ and promoting cell adhesion.^16^^,^^26^ Hence, targeting ENO1 is a promising approach for countering the chemoresistance developed during tumor growth. In nasopharyngeal carcinoma, the expression level of ENO1 was significantly upregulated in docetaxel-resistant tissues and cells, while inhibition of ENO1 restored tumor sensitivity to docetaxel.^86^ This has been further verified in a mouse endometrial carcinoma xenograft model. Compared with the control group, the shRNA-ENO1 group injected with cisplatin demonstrated more tumor growth inhibition.^85^ ENO1 has also been shown to induce the radio-resistance of tumor cells in the advanced stage of tumorigenesis. In a breast cancer xenograft model in mice, in which the mice were treated with radiation therapy, reduction of tumor volume and weight became more apparent in the siRNA ENO1 transfection group, indicating the strength of tumor cell sensitivity to radiation therapy mediated by the inhibition of ENO1 expression.^58^ Therefore, targeting ENO1 can improve the sensitivity of malignant tumors to chemotherapy and radiotherapy.

**Figure 3:**
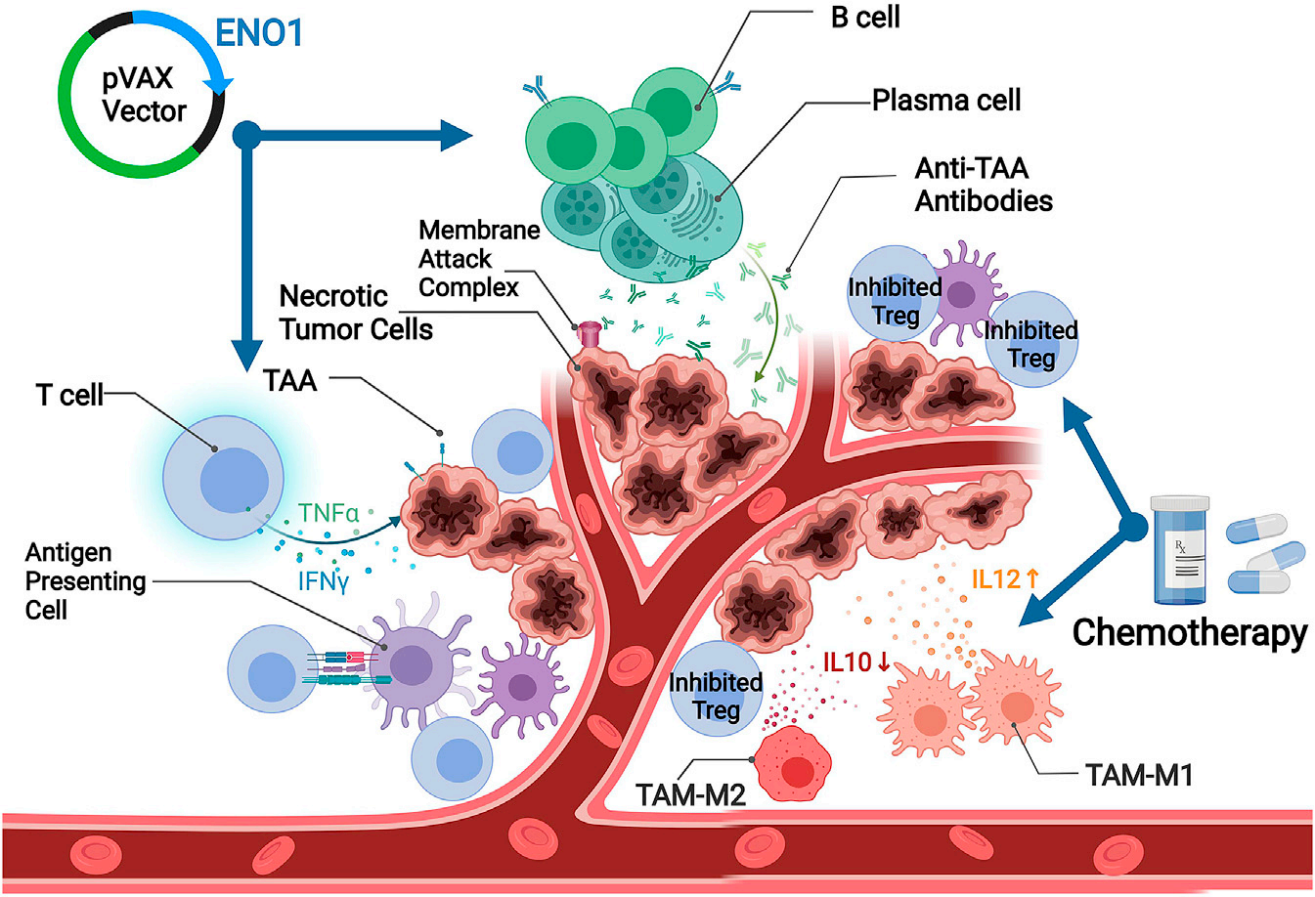
Schematic representation of the combinational effect of anti-ENO1 DNA vaccination and chemotherapy in pancreatic ductal adenocarcinoma mouse model The figure demonstrates that the vaccine induces various anti-tumor immune responses. Activated B cells and plasma cells produce anti-ENO1 antibodies. Membrane attack complex causes necrosis of tumor cells by inducing the complement-dependent cytotoxicity of tumor cells. Activated T cells release TNF-α and IFN-γ cytokines to damage tumor cells. Chemotherapy cyclophosphamide decreases the number of regulatory T cells (Tregs). There are two types of tumor-associated macrophage (TAM): M1-like phenotype TAM (M1-TAM), which releases anti-tumor interleukin-12 (IL-12) and M2-like phenotype (M2-TAM), which releases pro-tumor IL-10. Chemotherapy with GEM can upregulate TAM-M1 while downregulating TAM-M2.

The examination of immune responses to ENO1 has kindled the exploration of novel immunotherapeutic approaches. Anti-ENO1 monoclonal antibodies suppressed cell-associated plasminogen, matrix metalloproteinase activation, collagen and gelatin degradation, and cell invasion *in vitro*. Adoptive transfer of monoclonal anti-ENO1 antibodies to mice resulted in the accumulation of antibodies in subcutaneous tumors and inhibited the formation of tumor metastasis in the lung and bone *in vivo*.^31^ Similar results, whether *in vitro* or *in vivo*, have been validated in the highly aggressive PDAC, where anti-ENO1 monoclonal antibodies inhibited plasminogen-dependent invasion of human PDAC cells and their metastatic spreading in immunosuppressed mice was also inhibited.^32^ In pursuit of more sophisticated immunotherapy, Cappello et al.^87^^,^^88^ developed an anti-ENO1 DNA vaccine that can deliver multiple anti-tumor immune effects, including activation of anti-ENO1 Ab, induction of complement-dependent cytotoxicity of tumor cells, and the release of T lymphocyte cytokines (e.g., interferon-**γ** [IFN-**γ**], transforming growth factor α, and interleukin-17 [IL-17]). While the ENO1-targeting vaccine significantly reduced tumor progression in pre-clinical models, Cappello et al. admitted that the vaccine does not completely eradicate the tumor, which, after an initial growth inhibition, returns to proliferate again. Such limitation calls for further strategies for combinatorial treatments aiming at broadening and sustaining the anti-tumor immune response elicited by DNA vaccination. Cappello et al. applied cyclophosphamide (CTX) chemotherapy in conjunction with the DNA vaccine and achieved some success: CTX interferes with the mechanisms of tumor-induced immunosuppression. Tumor-associated macrophages (TAMs) in PDAC patients had a predominant M2-like immunosuppressive phenotype (M2-TAM), which is associated with a worse prognosis, except for patients who had received adjuvant CTX. Furthermore, chemotherapy influenced the interaction between macrophages and PDAC cells *in vitro*, as gemcitabine (GEM) enhanced the cytotoxic effect of M1-polarized macrophages (M1-TAM) while inhibiting the pro-tumor effect of M2-TAM. This was caused by the impact of GEM on macrophages, which showed an increase of M1-like markers (e.g., IL-12 and IFN-**γ**), and the downregulation of M2-like markers (e.g., IL-10)^89^ (Figure 3). In continuation of the effort made by Cappello et al.'s team, Mandili et al.^90^ have recently proven that treatment with GEM prior to ENO1 DNA vaccination unleashed CD4+ anti-tumor activity and strongly impaired tumor progression compared with mice that were vaccinated or GEM-treated alone. Indeed, combination strategies of vaccination together with inhibitors of immunologic checkpoints have been proven to be capable of overcoming tolerance and generating significant anti-tumor responses.

Despite recent advancements in biotechnologies, there still exist limitations in the current application of ENO1 as a tumor treatment target. Firstly, chemotherapy and medication treatments are given to patients in whom they might affect the expression of ENO1, leading to a potential bias. In addition, the relationship between ENO1 expression and patients with latter-stage cancers has not been fully understood, as ENO1 might show varied biological functions at different tumor stages. Efforts should be made to prepare prospective clinical trials to assess the prognostic value of ENO1 as a tumor marker in different stages of cancer and its effectiveness as a therapeutic target.

All things considered, ENO1-targeting immunotherapy has evolved from simple ENO1-depletion methods, such as Abs and small-molecule inhibitors, to more complex combinational therapies, such as applying DNA vaccines in conjunction with chemotherapy. Along the way, new anti-cancer features have been discovered: the inhibition of ENO1 counters chemoresistance. The combinational tumor therapies have shown success, and this leap of performance boost cannot be achieved by applying the treatments individually. In the near future, more ENO1-targeting drugs will emerge, with the propensity to be applied in combination with other treatment methods to receive optimal clinical outcomes.

While the existing technology of ENO1 therapy advances, there are two novel areas of ENO1 that deserve more attention. One is the more in-depth understanding of the physiological pathway of how ENO1 exteriorizes to the cell surface. Since the proteolytic effects of surface ENO1 contribute to cancer metastasis, knowledge of the ENO1 exteriorization signaling pathway can offer new oncotherapeutic targets that can smother ENO1-aided tumor metastasis in the cradle. In addition, combinational targeting of ENO1 linked with another antigen is believed to be a promising study scope in ENO1-involved oncotherapy. Bispecific antibodies (bsAbs) are artificially designed molecules that bind simultaneously to two different antigens, from which one is distributed at the tumor cell surface, and the other at an immune effector cell. Such dual antibody constructs can crosslink tumor cells and T cells. In contrast to ENO1 monoclonal Abs, bsAbs can be designed in such a way to involve the cytotoxic function of immune cells. This creates very promising prospects for a new generation of highly effective and much safer cancer therapies, providing durable anti-tumor immunity. In this scenario, more attention is required to support the safety usage in actual clinical applications and whether a synergistic effect can be achieved.

## Conclusions

In summary, ENO1 promotes cellular functions associated with tumor progression, including accelerated glycolysis, cancer cell proliferation, migration, invasion, drug resistance, and activation of oncogenic signaling pathways. Contributing to several hallmarks of cancer, ENO1 can doubtlessly be stamped as an oncoprotein, especially in consideration of its function in deregulating glucose metabolism and, consequently, triggering tumor proliferation and metastasis. The localization of ENO1 on the cell surface makes it an ideal prognostic and diagnostic cancer biomarker. More importantly, the consistent correlation between overexpression of ENO1 on multiple cancer types and worse clinical outcomes suggests its key role in activating oncogenic signaling pathways and positions it to be an optimal therapeutic target. Although glycolysis is an ideal oncotherapeutic targeting pathway, it is ubiquitous in all cells. Thus, how to initiate anti-tumor activity while avoiding the danger of shutting down glycolysis remains a challenging problem. Ultimately, the pre-clinical success of using combinatorial therapy of novel ENO1-targeting inhibitors along with traditional chemotherapy sheds light of hope to patients who have borne “cureless” cancers.
